# Can Ipilimumab restore immune response in advanced NSCLC after progression on anti‐PD‐1/PD‐L1 agents?

**DOI:** 10.1111/1759-7714.13502

**Published:** 2020-06-16

**Authors:** Michal Sternschuss, Nir Peled, Aaron M. Allen, Elizabeth Dudnik, Ofer Rotem, Noga Kurman, Omer Gal, Hiba Reches, Alona Zer

**Affiliations:** ^1^ Davidoff Cancer Center Rabin Medical Center Petah Tikva Israel; ^2^ Sackler School of Medicine Tel Aviv University Tel Aviv Israel; ^3^ The Legacy Heritage Oncology Center Soroka Medical Center Beer‐Sheva Israel; ^4^ Ben Gurion University of Negev Beer‐Sheva Israel

**Keywords:** Immune‐related adverse events, ipilimumab, nivolumab, NSCLC

## Abstract

Anti‐PD‐1/PD‐L1 agents play a crucial part in the treatment of non‐small cell cancer (NSCLC) demonstrating improved overall response rate (ORR) and overall survival (OS). Recent studies evaluating combination treatment with anti‐PD‐1 and anti‐CTLA‐4 suggests improved outcome but also increased toxicity. Evidence is scarce regarding subsequent treatment with immune checkpoint inhibitors (ICPI) after progression on anti‐PD‐1/PD‐L1. A total of 15 patients were treated with a combination of anti‐PD1 agent and ipilimumab after confirmed progression of disease on anti‐PD1/PDL1 alone during 2017. Clinical data were retrieved retrospectively. Disease control rate (DCR) was defined as partial response (PR) or stable disease (SD). The overall DCR was 33.3% (*n* = 5); two patients with PR and three patients with SD, three of whom had prior documented disease control on anti‐PD1. The immune‐related adverse event (irAE) rate was 40% (*n* = 6); two patients had grade 3 AE and one patient died of pneumonitis. While the median time to progression was two months (range 0.5–16), four of the five patients with PR/SD experienced durable benefit for 8–16 months. This small retrospective cohort of heavily pretreated unselected patients suggests ipilimumab might reboost the immune response in patients with advanced NSCLC following progression of disease on anti‐PD1 therapy, while delaying exposure to the higher toxicity rates associated with upfront combination therapy. This strategy should be explored prospectively.

## Introduction

Immune checkpoint inhibitors (ICPI) play an increasingly crucial role in the treatment paradigm of metastatic non‐small cell lung cancer (mNSCLC) and are now considered the standard of care in both first and advanced lines setting, demonstrating improved objective response rate (ORR) and overall survival (OS) compared with traditional chemotherapy regimens.

Different approaches are being evaluated to maximize treatment efficacy. One approach is combination of inhibitors targeting different immune checkpoints. Anti‐programmed death 1 (PD‐1) and anticytotoxic T cell lymphocyte‐4 (CTLA‐4) antibodies have distinct, complementary mechanisms of action and thus, the combination may improve antitumor immunity as demonstrated in other malignancies. A phase 1 study evaluating combination therapy in unselected treatment naïve mNSCLC patients suggested improved ORR and durable responses, at a range of 33%–37% grade 3/4 immune‐related adverse events (irAEs).^1^ A more recently published phase 3 trial compared combination immunotherapy to standard chemotherapy in patients with a high tumor mutational burden (TMB) and found improved progression‐free survival (PFS) and ORR.^2^ This trial also reported a grade 3/4 irAE rate of 31.2% as compared with 7%–26% in the major randomized control trials of single agent anti‐PD‐1/PD‐L1.

Evidence is scarce regarding subsequent treatment with ICPI after progression on initial treatment with ICPI. Small cohorts and case reports suggest clinical benefit with the rechallenge approach.^3^, ^4^, ^5^ A study in melanoma patients reported similar ORR to ipilimumab single agent after progression on anti‐PD‐1 as was reported in treatment naïve patients, regardless of previous response to anti‐PD‐1.^6^ A recently published abstract reported a 60% disease control rate in melanoma patients treated with combination pembrolizumab and ipilimumab after progression on anti‐PD‐1.^7^

Here, we report a small cohort of mNSCLC patients treated with combination anti‐PD‐1 and anti‐CTLA‐4 after progression on anti‐PD‐1/PD‐L1 agents.

## Methods

All off‐label ipilimumab administrations in a single tertiary center were retrieved through the institutional review board records (single patient requests). A total of 21 patients with mNSCLC were identified between January and December 2017. Clinical data including baseline characteristics, previous and current treatments, response evaluations and toxicity reports were retrieved retrospectively through electronic medical records.

Disease control rate (DCR) was defined as complete response (CR), partial response (PR) or stable disease (SD) extracted from clinical notes and adapted from RECIST criteria. Time to progression was defined as interval between first dose to documented radiological progression or death. TMB was adopted from next generation sequencing reports when available, high TMB defined as >10mut/Mb. irAEs were assessed according to Common Terminology Criteria for Adverse Events version 4.0.

This study was approved by the Rabin Medical Center Ethical committee and the Institutional Review Board.

## Results

Of 21 patients with mNSCLC who were treated with a combination of anti‐PD1 agent and ipilimumab, in 15 ipilimumab was initiated after confirmed progression on anti‐PD1/PDL1 alone.

Patients' characteristics are described in Table 1. The patients were all male, median age 67 (range 53%–87), 80% of patients were past or current smokers. Tumor histology consisted of adenocarcinoma, adenosquamous and NSCLC‐NOS. Molecular analyses for all patients were negative for *EGFR* and *ALK* aberrations, one patient had a *MET*‐amplified tumor and two patients had high TMB. PD‐L1 expression was variable and data was not available in almost half of the patients due to inadequate tissue samples. A total of 86.7% of patients received at least one line of systemic therapy before anti‐PD‐1/PDL‐1 treatment, 12 of them received platinum‐based doublet chemotherapy and one patient with *MET* amplified tumor received crizotinib. A total of 86.7% of patients received palliative radiation therapy at some point of their treatment.

**Table 1:** Patients' characteristics

Patients' characteristics	*N* = 15 (%)	
Sex	Male (15), Female (0)	
Median age (range)	67 years (53–87)	
Smoking history	Yes	12 (80%)
	no	3 (20%)
Histology	Adenocarcinoma	12 (80%)
	Squamous	1 (6.7%)
	Adenosquamous	1 (6.7%)
	NSCLC‐NOS	1 (6.7%)
PD‐L1 expression	>50%	2 (13.3%)
	1–50%	4 (26.7%)
	<1%	2 (13.3%)
	Unknown	7 (46.7%)
Line of treatment with anti‐PD1/PDL1	First	2 (13.3%)
	Second	12 (80%)
	Third	1 (6.7%)
Best response to anti‐PD1/PDL1	PR	4 (26.6%)
	SD	3 (20%)
	PD	8 (53.4%)
Median time to progression on anti‐PD1 (range)	2 months (1–17)	

**Table 2:** Immune‐related adverse effect (irAE) rate

Toxicity	All grades	Grade 1/2	Grade 3/4	Grade 5
Arthralgia and myalgia	2 (13.3%)†	2 (13.3%)	‐	‐
Diarrhea	2 (13.3%)	‐	2 (13.3%)	‐
Pneumonitis	2 (13.3%)	1 (6.7%)	‐	1 (6.7%)
Dermatitis	1 (6.7%)†	1 (6.7%)	‐	‐

† One patient had both grade 1 arthralgia and grade 1 dermatitis.

### Previous anti‐PD‐1/anti‐PD‐L1 treatment

One patient (6.7%) received atezolizumab (anti‐PD‐L1), three (20%) received pembrolizumab (anti‐PD‐1) and 11 (73.3%) received nivolumab (anti‐PD‐1). The overall DCR for single agent anti PD‐1/PD‐L1 was 46.6% (*n* = 7): four patients with PR and three patients with SD (one patient with PR received combination immunotherapy‐chemotherapy). All 15 patients had documented progression of disease according to the clinic records and imaging reports. The median time to progression was two months (range 1–17 months).

irAE were documented in 46.6% (*n* = 7) of the patients and were all G1/2 which did not require discontinuation of the treatment.

### Combination therapy

All patients received at least one dose of combination therapy with ipilimumab 1 mg/kg every four weeks with either nivolumab 3 mg/kg every two weeks (*n* = 11) or pembrolizumab 2 mg/mg every three weeks (*n* = 4). Median number of ipilimumab cycles was three (range 1–9).

**Figure 1:**
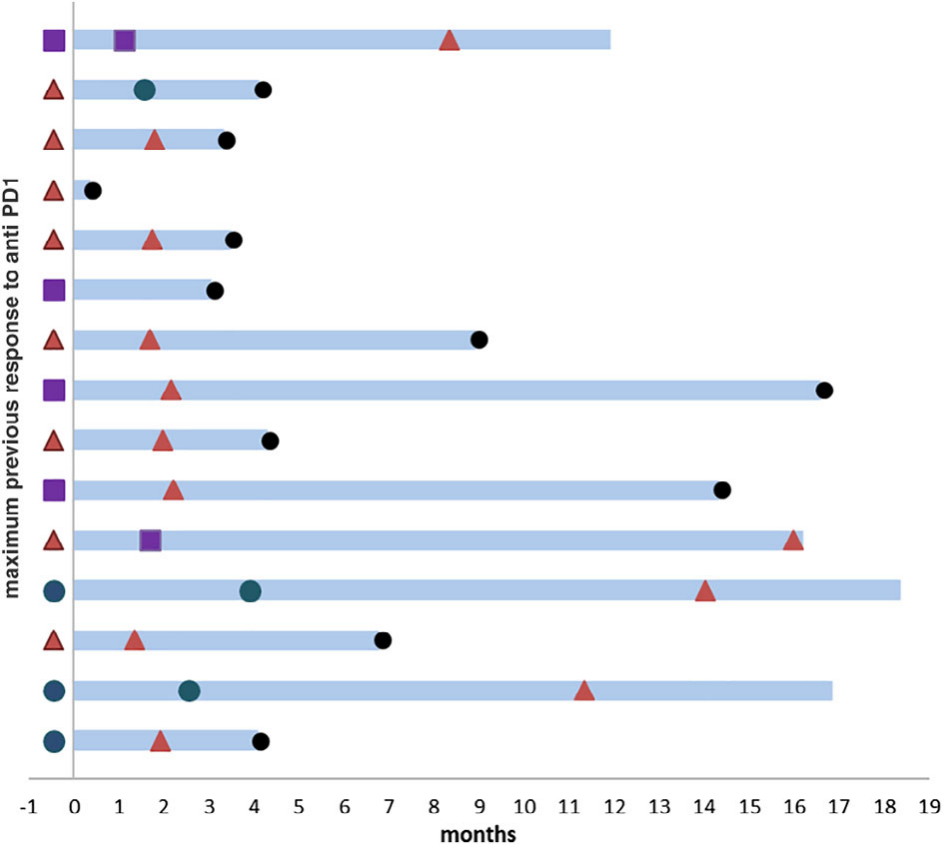
Response to combination therapy. Swimmers plot displaying individual patients' outcome during follow up since initiation of combination therapy. Each bar represents one subject in this study. (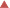) PD, (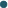) SD, (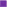) PR, (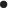) Death.

Response to treatment is described in Figure 1. The overall DCR was 33.3% (*n* = 5); two patients with PR and three patients with SD. While the median time to progression was two months (range 0.5–16), four of the five patients with PR/SD experienced durable benefit for eight, 12, 14 and 16 months before progression. Three of them showed previous clinical benefit from single agent ICPI for 6–17 months before progression. Of these patients, one patient with *MET*‐amplified tumor also had PD‐L1 expression higher than 50% as well as high TMB, two patients had PD‐L1 expression of 1%–25% and two had less than 1% expression. The median pack year smoking history for the benefiting patients was 40 years (range 0–80) as compared with 30 years (range 0–120) for the entire cohort.

After progression, two patients continued treatment with anti‐PD‐1 with palliative radiation to symptomatic progression sites. Only three patients received subsequent treatment. The median OS from initiation of ipilimumab was 5.5 months (range 0.5 ‐ not reached).

All grade irAE rate was 40% (*n* = 6); two patients had grade 3 diarrhea requiring steroids and discontinuation of treatment and one patient died of grade 5 pneumonitis. irAE rates are described in table 2. Notably, all patients with grade 3–5 irAEs displayed PR or SD.

## Discussion

ICPI have revolutionized the treatment and prognosis of patients diagnosed with mNSCLC. Despite major advances in terms of patients' outcome, maximizing efficacy of treatment with ICPI while taking into account the toxicity profile is one of the main challenges facing medical oncologists today. In this small retrospective cohort, we report a DCR of 33.3% with a combination of anti‐CTLA‐4 and anti‐PD‐1 in 15 patients who previously progressed on anti‐PD‐1/PD‐L1. While the DCR is lower than reported in previous trials of combination ICPI,^1^, ^2^ the patient population in our cohort consisted of heavily pretreated unselected patients.

Multiple clinical trials demonstrated that while combination of anti‐CTLA‐4 and anti‐PD‐1 show clinical benefit superior to single agent anti‐PD‐1, the irAE rate is significantly higher including treatment related deaths.^1^, ^2^ This current observation suggests that the clinical benefit of combination ICPI might still be achieved after progression on anti‐PD‐1/PD‐L1 agents, thus delaying the exposure to higher rates of irAEs and potentially sparing them altogether from the subgroup of patients with durable response to single agent ICPI. Our results also align with reported rates of irAEs, unfortunately including one treatment related death, emphasizing the undeniable toxicity of combination treatment.

Another point to consider is the resistance mechanisms to ICPI as primary and acquired resistance represent a significant clinical challenge and the treatment options thereafter are limited. Several mechanisms of resistance have been suggested, including upregulation of alternative immune checkpoints such as T cell immunoglobulin mucin‐3 (TIM‐3) or CTLA‐4 and mutations in pathways involved in interferon‐receptor signaling and antigen presentation.^8^, ^9^, ^10^ One study found that the therapeutic anti‐PD‐1 antibodies were still bound to the T cells at the time of disease progression.^9^ This observation supports continuing treatment with anti‐PD‐1 agent beyond progression while adding a second agent blocking an alternative pathway.

Our main limitation is the small sample size and retrospective nature of this study as well as limited data regarding PD‐1 expression and tumor mutational burden.

Many questions remain unanswered in terms of optimal treatment sequence and patient selection. Prospective data is needed to further explore a potential role for ICPI‐combination beyond PD in mNSCLC.

In conclusion, this small retrospective cohort suggests ipilimumab might enhance and reboost immune response in patients with advanced NSCLC progressing on anti‐PD‐1/PD‐L1 therapy, while delaying exposure to the higher rates of irAE associated with upfront combination therapy. This strategy should be explored prospectively.

## Disclosure

No authors report any conflict of interest.
